# Integrated Pharmacophore Modeling, Molecular Docking, and Molecular Dynamics Simulations Accelerate the Discovery of Novel PDE1 Inhibitors with Potential for the Treatment of Idiopathic Pulmonary Fibrosis

**DOI:** 10.3390/molecules31101731

**Published:** 2026-05-19

**Authors:** Xin-Lin Cai, Zhao-Hang Xue, Shu-Jin He, Wei-Hao Luo, Run-Duo Liu, Qian Zhou, Chen Zhang

**Affiliations:** 1 School of Chemistry and Chemical Engineering, Guangdong Pharmaceutical University, Zhongshan 528458, China; 2112440011@stu.gdpu.edu.cn (X.-L.C.); 2112340005@stu.gdpu.edu.cn (Z.-H.X.); 2112542076@stu.gdpu.edu.cn (S.-J.H.); 2112373045@stu.gdpu.edu.cn (W.-H.L.); 2 School of Pharmaceutical Sciences, Sun Yat-sen University, Guangzhou 510006, China; liurd3@mail2.sysu.edu.cn; 3 Key Laboratory of Tropical Biological Resources of Ministry of Education and Hainan Engineering Research Center for Drug Screening and Evaluation, School of Pharmaceutical Sciences, Hainan University, Haikou 570228, China

**Keywords:** phosphodiesterase-1, pharmacophore modeling, molecular dynamics, virtual screening

## Abstract

Phosphodiesterase-1 (PDE1) represents an attractive target for the treatment of idiopathic pulmonary fibrosis (IPF). However, the limited chemical diversity of current PDE1 inhibitors has hindered the development of potential anti-IPF drugs, primarily due to an ambiguous understanding of interactions between inhibitors and PDE1. Herein, we report an integrated virtual screening strategy containing pharmacophore modeling, molecular docking, and molecular dynamics simulations, which markedly accelerated the discovery of novel PDE1 inhibitors. Enzymatic assays identified eleven active compounds with moderate inhibition from twenty-six purchased candidates, encompassing nine distinct scaffold types. Notably, 6484-0008 and 6484-0032 exhibited more than 50% inhibition at a concentration of 1 μM. Hydrogen bond analysis and residue-based energy decompositions revealed key recognition mechanisms involving crucial residues Gln421, His373, and Phe424, as well as the unique Thr271 in the flexible H-loop region, providing insights for the rational design of inhibitors with enhanced potency.

## 1. Introduction

Idiopathic pulmonary fibrosis (IPF) is a chronic, progressive interstitial lung disease with variable courses. If left uncontrolled, pulmonary fibrosis can ultimately lead to progressive dyspnea and even death, with a median survival time of only 2–4 years after diagnosis. The etiology of pulmonary fibrosis is complex, including but not limited to genetic and environmental risk factors [1,2,3]. Pure anti-inflammatory treatment has poor efficacy in treating pulmonary fibrosis. Conventional medications used to treat pulmonary fibrosis are pirfenidone and nintedanib, which exert their effects by targeting angiogenesis-related receptor tyrosine kinases and TGF-β and its downstream pathways, respectively. However, these drugs can only delay the disease progression and have non-negligible adverse reactions, such as rash, nausea, and diarrhea [4,5,6]. Clinically, there is an urgent need for targeted therapeutic drugs for IPF with new mechanisms of action and fewer side effects.

Cyclic nucleotides, particularly adenosine 3′,5′-cyclic phosphate (cAMP) and guanosine 3′,5′-cyclic phosphate (cGMP), serve as crucial second messengers regulating a wide range of cellular processes, including proliferation, inflammation, and fibrosis [7,8]. The intracellular levels of these nucleotides are tightly controlled by phosphodiesterases (PDEs), a superfamily of metal-dependent hydrolases that catalyze their degradation [9,10]. There are plenty of drugs developed targeting PDEs, including blockbuster drugs such as sildenafil citrate and apremilast [11,12,13]. Among the eleven PDE families, PDE1 is unique in that it is activated by calcium-calmodulin (CaM), thereby linking calcium signaling to cyclic nucleotide homeostasis [14,15]. PDE1 can hydrolyze both cAMP and cGMP and exists in three isoforms. PDE1A is the main PDE1 isoform in vascular smooth muscle cells (VSMCs), where its inhibition elevates intracellular cGMP levels, thereby promoting cGMP-mediated vasorelaxation [16,17]. PDE1B is the dominant isoform present in medium spiny neurons of the striatum, and inhibiting PDE1B can boost long-term potentiation (LTP) and anti-Parkinsonian effects [18,19]. PDE1C is primarily found in cardiomyocytes and synthetic VSMCs. In synthetic VSMCs, the expression of PDE1C is upregulated, promoting their state of proliferation or migration [20,21]. In lung fibroblasts, the predominant PDE activity is attributed to PDE1, 4, and 5 [22]. The recent approval of nerandomilast (BI 1015550), a PDE4 inhibitor, marks a major milestone in IPF therapeutics, as it is the first new drug to demonstrate efficacy in phase III trials in over a decade, reinforcing PDEs as tractable targets for fibrosis intervention [23]. We have recently identified that inhibiting PDE1 prevents the phenotypic conversion of fibroblasts to myofibroblasts induced by transforming growth factor-β (TGF-β), a hallmark event in the pathogenesis of fibrosis [24]. Meanwhile, in a bleomycin-induced pulmonary fibrosis rat model, PDE1 inhibition can alleviate pulmonary fibrosis by inhibiting the TGF-β/Smad and mitogen-activated protein kinase (MAPK) signaling pathways, thereby validating the potential of PDE1 as a therapeutic target for IPF [25].

**Figure 1:**
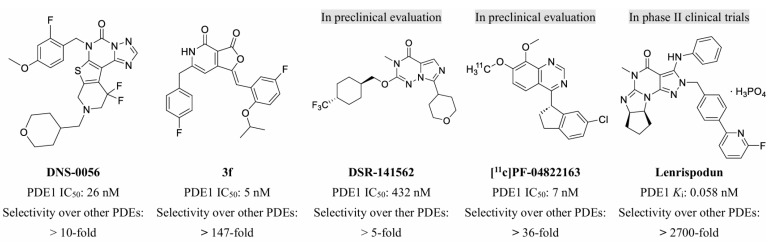
Representative molecules selectively inhibiting PDE1.

As a therapeutically attractive target, PDE1 has garnered considerable attention, spurring the development of its inhibitors (Figure 1) [26,27,28,29]. Among reported inhibitors, Lenrispodun (ITI-214) shows picomolar inhibition with high selectivity and has advanced to Phase II clinical trials for heart failure and Parkinson’s disease [26]. However, the diversity of current PDE1 inhibitors remains limited, particularly with regard to selective ones, primarily due to the high conservation of catalytic domains across different PDE families [9,10]. The absence of a detailed structure–activity relationship (SAR) for PDE1 inhibitors has substantially hindered structure-based rational design efforts. We have recently reported a novel strategy for the design of PDE1 inhibitors, which achieve remarkable selectivity against other PDE families by targeting the H-loop region of PDE1 [25]. Based on high-resolution crystal structures of enzymatic catalytic domains in complex with various small molecules, molecular modeling and receptor-based virtual screening have offered powerful and cost-effective approaches for identifying novel inhibitors from compound libraries [30,31,32]. However, the application of this approach to the efficient discovery of PDE1 inhibitors has rarely been reported.

**Figure 2:**
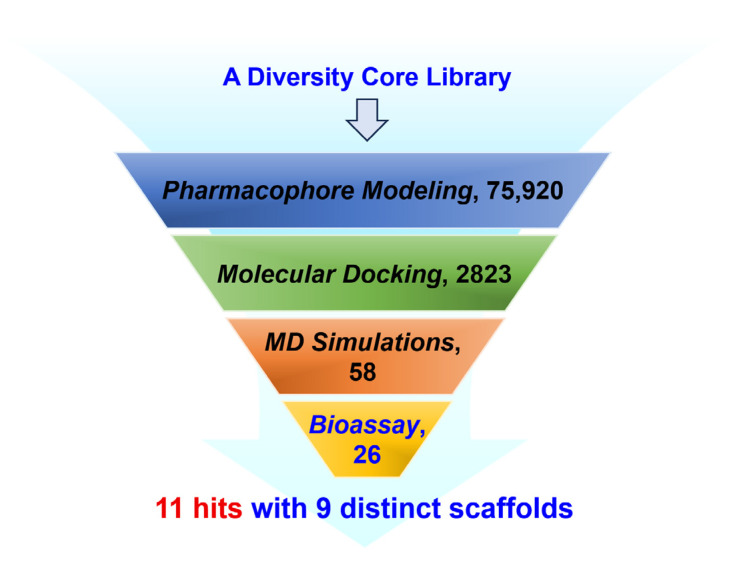
Workflow of virtual screening for the identification of novel PDE1 inhibitors.

In this study, we aimed to discover novel PDE1 inhibitors with diverse scaffolds from a commercial compound library. To achieve this, we performed virtual screening that integrated pharmacophore modeling, molecular docking, and dynamics simulations. Subsequently, the PDE1 inhibitory activity of the purchased compounds was evaluated through in vitro enzymatic assays. Through this integrated approach, we identified 11 PDE1 inhibitors with novel structural features from 26 compounds, yielding a hit rate of 42% (Figure 2). These results expand the existing chemical diversity of PDE1 inhibitors and provide new avenues for further optimization as tool compounds in pulmonary fibrosis research.

## 2. Results

### 2.1. Pharmacophore Modeling and Virtual Screening Substantially Decrease the Scale of the Initial Compound Library

#### 2.1.1. Ligand-Based Pharmacophore Modeling

**Figure 3:**
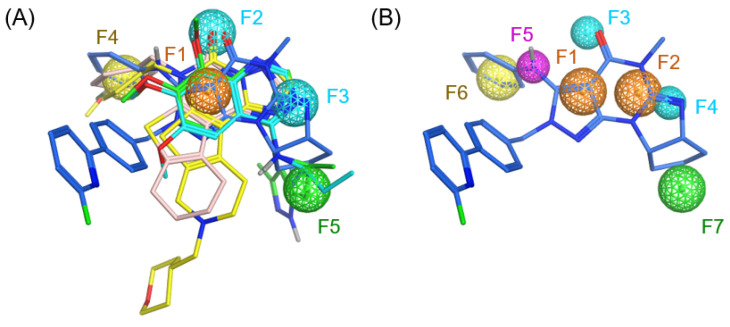
(**A**) The initial pharmacophore model derived from five inhibitors comprises five features (F1–F5). F1 represents an aromatic or aromatic-like ring center rich in π electrons. F2 and F3 denote hydrogen bond acceptors. F4 corresponds to an aromatic or hydrophobic ring center. F5 represents a hydrophobic center. (**B**) The refined pharmacophore model comprises seven features (F1–F7). F1 and F2 represent aromatic or aromatic-like ring centers, each with a radius of 1 Å. F3 and F4 denote hydrogen bond acceptors, each with a radius of 0.7 Å. F5 corresponds to a linking atom with a radius of 0.7 Å. F6 represents an aromatic or hydrophobic ring center with a radius of 1 Å. F7 denotes a hydrophobic center with a radius of 1 Å.

Five crystal structures of PDE1 catalytic domains in complex with small-molecule ligands (PDB IDs: 4NPV, 4NPW, 5B25, 5UOY, and 5UP0) [26,27,33] were superimposed based on amino acid sequence alignment, and all bound ligands were extracted to define the pharmacophore features. As observed, all ligands share a multi-fused aromatic ring system bearing a carbonyl or methoxyl group at a conserved position, along with an imino nitrogen at a corresponding location on the same ring. Additional common features include a phenyl ring or a methyl group adjacent to the aforementioned carbonyl or methoxyl moiety, as well as a hydrophobic ring or a branched alkyl chain near the imino nitrogen. Based on the above analysis, a preliminary pharmacophore model was established, and the radius of each feature was initially set to 1 Å (Figure 3A). This model incorporated critical features associated with the unique hydrophobic pocket and the flexible H-loop region, which to our knowledge have not been explicitly considered in previous virtual screening campaigns targeting PDE inhibitors.

To assess the reliability of the pharmacophore model, a test set consisting of known PDE1 inhibitors and inactive compounds was constructed and employed for model refinement. Screening of the test set yielded a true positive rate of 0.85 (seventeen out of twenty inhibitors) and a false positive rate of 0.01 (twenty-five out of two thousand inactive compounds). In addition, a Goodness of Hit (GH) score greater than 0.5 indicates acceptable model quality [34]. The calculated GH score of 0.51, together with an enrichment factor of 40.88 at 1% database screening, confirms that the model is sufficiently reliable for subsequent virtual screening (Table 1).

#### 2.1.2. Rapid Screening Using Established Pharmacophore Model

A diversity-focused core library consisting of 75,920 compounds from the ChemDiv chemical libraries has been selected for virtual screening. The molecular weights of most compounds in this library range from 250 to 450, rendering them suitable as starting points for further modification. For each compound, up to 250 conformations were generated and screened against the refined pharmacophore model. As a result, 2823 compounds passed the initial screening and were forwarded for subsequent molecular docking.

### 2.2. Molecular Docking and Visual Inspection Identify Optimal Binding Poses

#### 2.2.1. Validation of the Docking Protocol

Before molecular docking of the library, five known inhibitors, which were previously used for pharmacophore modeling, were re-docked into the catalytic domain of PDE1 (PDB ID: 5B25). For all five inhibitors, the resulting poses consistently reproduced a hydrogen bond with Gln421 and π-π interactions with Phe424. Additionally, four of the five inhibitors also formed a hydrogen bond with His373. Overall, the docking poses faithfully reproduced the known binding patterns to PDE1, with the top-ranking poses exhibiting a root-mean-square deviation (RMSD) of less than 1.5 Å from the original crystal poses, indicating that the docking protocol is reliable.

#### 2.2.2. Screening for Optimal Binding Poses

**Figure 4:**
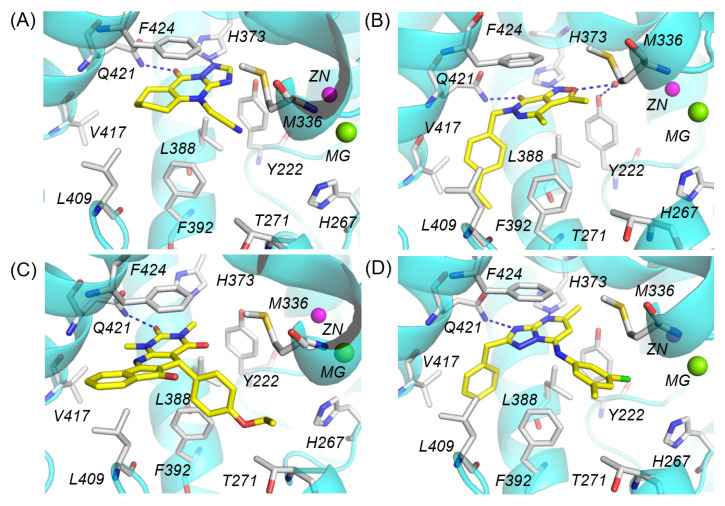
Representative binding poses of retained compounds (in yellow sticks) with PDE1 (in cyan ribbons and grey sticks). Key interactions include hydrogen bonds with Gln421 and/or His373, as well as π-π interactions with Phe424. (**A**) Compound 8012-2471 displays no apparent contacts with the Q2 pocket or H-loop region. (**B**) Compound F279-0419 forms a water-mediated hydrogen bond with Tyr222, while its 4-vinylbenzyl group occupies the Q2 pocket. (**C**) Compound 3389-0956 extends its 4-ethoxyphenyl group towards the H-loop region. (**D**) Compound D245-0568 simultaneously interacts with the Q2 pocket and H-loop region through a benzyl and 3-chloro-5-methylphenyl group. Intermolecular hydrogen bonds are indicated by blue dashed lines.

Molecular docking was performed on all compounds that passed the pharmacophore screening. Of the 2823 compounds, 2672 were successfully docked into the catalytic domain. A small number of exceptionally large molecules could not be physically accommodated within the protein pocket and generated no valid docking poses due to severe steric clashes with nearby residues. In the first round of visual inspection, such interactions were prioritized: direct hydrogen bonds with either Gln421 or His373 and π-π interactions with Phe424. Compounds lacking these interactions or causing obvious steric hindrance with PDE1 were removed at this stage. In the second round, compounds were preferentially retained if they possessed either a large hydrophobic group entering the Q2 pocket (composed of Leu388, Phe392, Leu409, and Val417) or a substituent extending toward the H-loop region (specifically His267 and Thr271) (Figure 4 and Figure S1). Additionally, derivatives sharing the same core but featuring various substituent groups were kept as much as possible. As a result, 81 compounds representing 29 distinct cores were retained. Among these, 64 representative compounds with diverse substituent groups were selected for subsequent simulations.

### 2.3. Molecular Dynamics-Based Virtual Screening Narrows the Hit List to Fewer than 30 Compounds

#### 2.3.1. Screening of Pan Assay Interference Compounds

Before molecular dynamics (MD) simulations, pan-assay interference compounds (PAINS) should be excluded since they might interfere with the enzymatic assays [35]. The online tool ChemFH [36] was utilized for the screening, leaving 58 compounds for further study.

#### 2.3.2. Molecular Dynamics Simulations of Protein-Ligand Complexes

For each protein–ligand system (protein PDB ID: 5B25), a 100 ns MD simulation was conducted in an explicit water environment. Subsequently, the RMSD values of the backbone atoms in PDE1 were calculated for each system, and only those with maximum RMSD values below 2.5 Å for the protein backbone atoms—indicative of stable binding modes—were retained (Figure S2). The binding free energies were then estimated over the final 2.5 ns of simulation using the Molecular Mechanics—Poisson Boltzmann (Generalized Born) Surface Area (MM-PBSA and MM-GBSA) methods [37], and the compounds were ranked accordingly to select the optimal ones (Table 2 and Table S1). As a result, fewer than 30 compounds were retained.

### 2.4. Enzymatic Assays Confirmed Moderate PDE1 Inhibitory Activity for 11 Compounds

A total of 26 compounds were purchased and evaluated for inhibitory activity against PDE1 by means of enzymatic assays. As a result, 11 compounds were validated as moderate PDE1 inhibitors, demonstrating inhibition rates beyond 50% at a concentration of 10 μM, yielding a high hitting accuracy of 42%. Notably, compounds 6484-0008 and 6484-0032 exhibited superior potency, achieving greater than 50% inhibition at a reduced concentration of 1 μM (Table 2). Further enzymatic assays revealed that all compounds except for compound 8020-2441 displayed inhibition rates below 50% against PDE9 at a concentration of 50 μM, corresponding to a selectivity fold of greater than 5 (Table S1).

## 3. Discussion

### 3.1. Nine Novel Scaffolds Were Identified in PDE1 Inhibitors

**Figure 5:**
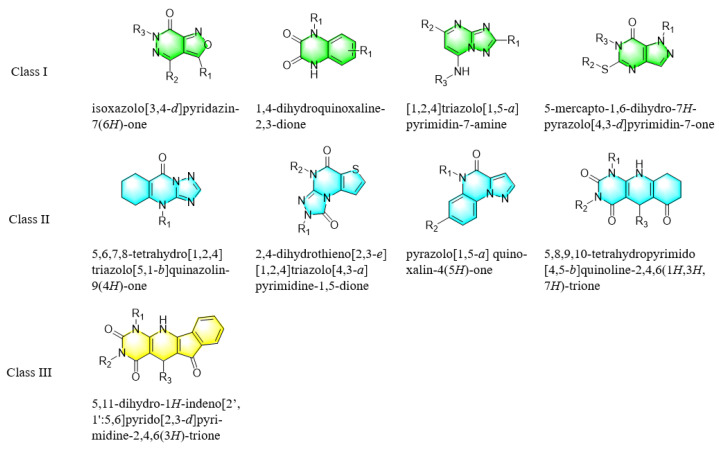
Nine novel scaffolds identified among the active hits and organized into three structural classes.

A systematic comparison of our hits with reported hits in terms of scaffold chemotypes, potency, and selectivity profile underscores that our compounds expand the currently limited PDE1 chemotypes and offer new starting points for SAR exploration (Table S3) [27,33,38]. Relative to reported PDE1 inhibitor chemotypes, the eleven active hits displayed nine structurally distinct scaffolds, which were organized into bicyclic (C200-4020, 8020-2441, D245-0568, F279-0419), tricyclic (8012-24715223-1356, L397-0566, P300-1479), and tetracyclic (6484-0008, 6484-0032, 3389-0956) core structures (Figure 5).

### 3.2. Hydrogen Bond Analysis Validated Critical Interactions of Gln421 and His373 with the Inhibitors

**Figure 6:**
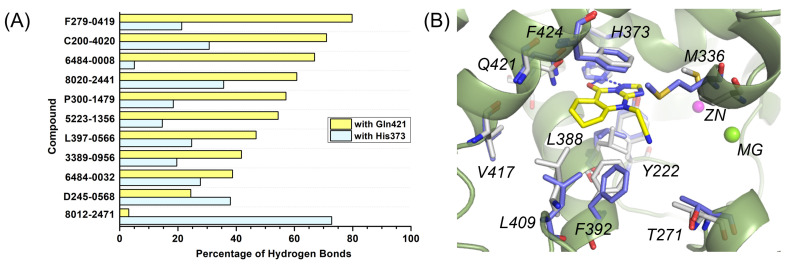
(**A**) Percentage of hydrogen bonds formed during MD simulations. (**B**) The binding mode of 8012-2471 (in yellow sticks) within PDE1 (in olive ribbons) following 20 ns MD simulations (key residues as blue sticks), superimposed with the initial structure (key residues as grey sticks). Hydrogen bonds are indicated by blue dashed lines.

A comprehensive hydrogen bond analysis was conducted for all identified hits based on MD simulation results. Statistical data revealed that nine of the eleven inhibitors established stable hydrogen bonds with Gln421 within the pocket, with occupancies during the first 20 ns approaching or exceeding 40% (Figure 6A and Table S2). In contrast, 8012-2471 formed a stable hydrogen bond exclusively with His373, a residue unique to PDE1 among the PDE superfamily. Destabilization of the hydrogen bond between Gln421 and 8012-2471 may result from steric clashes involving the cyclohexyl group, prompting a conformational shift in Gln421 (Figure 6B). Notably, the majority of Gln421-binding inhibitors simultaneously interacted with His373. This dual residual recognition mode likely underlies the observed selectivity against PDE9 (Table S1). Furthermore, geometric analysis indicated that inhibitors generally formed better hydrogen bonds with Gln421 than with His373, as evidenced by average hydrogen bond angles closer to the ideal 180° for the former (Table S2).

### 3.3. Energy Decomposition Explained Activity Differences in Three PDE1 Inhibitors

In the predicted binding energy intervals below −40 kcal/mol or above −20 kcal/mol, none of the tested compounds exhibited PDE1 inhibitory activity (0% enrichment in both intervals). Within the −40 to −30 kcal/mol range, 8 out of 15 compounds were found to be active (53% enrichment). Within the −30 to −20 kcal/mol range, 3 out of 8 compounds showed inhibitory activity (38% enrichment). Collectively, these results reveal a clear energy–activity relationship: active compounds are predominantly enriched within the moderately favorable binding energy window (−40 to −20 kcal/mol), whereas neither the most favorable (<−40 kcal/mol) nor the least favorable (>−20 kcal/mol) intervals yielded active hits, suggesting that this intermediate energy range may serve as an optimal priority filter for future screening campaigns.

**Figure 7:**
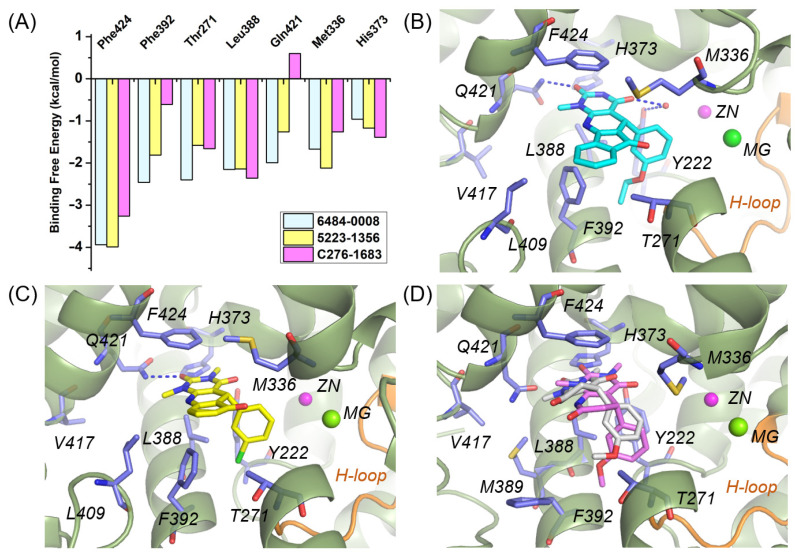
(**A**) Residue-based decomposition of the binding free energies for the three complexes. (**B**–**D**) Final binding poses of 64840008 (cyan), 5223-1356 (yellow), and C276-1683 (pink) within PDE1 (in olive ribbons and blue sticks) after 20 ns MD simulation. Superimposed initial conformation of C276-1683 is colored grey. The H-loop region is colored orange. Hydrogen bonds are indicated by blue dashed lines.

Among the active hits identified from screening, five compounds (6484-0008, 5223-1356, 6484-0032, 3389-0956, and C276-1683) share similar binding patterns with PDE1, yet exhibit disparate inhibitory activities (Table 2 and Figure S1). Overall, compounds bearing a tetracyclic core (6484-0008, 6484-0032, and 3389-0956) exhibited comparable activity to their tricyclic counterpart 5223-1356, whereas C276-1683, with a bicyclic core, showed markedly reduced potency. To explain these differences, residue-based energy decomposition was performed on the complexes of 6484-0008, 5223-1356, and C276-1683 with PDE1 using the MM-GBSA method over the MD trajectories from 17.5 to 20 ns. The results (Figure 7A) focused on seven residues contributing significantly (<−1.0 kcal/mol) to binding: Phe424, Phe392, Thr271, Leu388, Gln421, Met336, and His373. Notably, Phe424, Phe392, and Leu388 provided major contributions to the total binding free energies of 6484-0008 and 5223-1356, underscoring the importance of π-π and hydrophobic interactions in inhibitor recognition. Thr271 in the flexible H-loop region represents another critical residue. Inhibitor binding may induce the H-loop’s conformational changes that optimize these interactions (Figure 7B,C). Strikingly, the marked energy difference between C276-1683 and 6484-0008 mainly originated from Gln421 (−1.99 and 0.6 kcal/mol) and Phe392 (−2.46 and −0.61 kcal/mol), corresponding to a 10-fold potency loss. The MD-based pose of C276-1683 revealed substantial positional deviation from its initial docking conformation (Figure 7D), leading to a loss of hydrogen bonds with crucial Gln421 and π-π interactions with Phe392, consistent with the energy decomposition analysis.

## 4. Materials and Methods

### 4.1. Molecular Modeling and Virtual Screening

In the workflow, molecular modeling based on known potent inhibitors resulted in an efficient pharmacophore model. Molecules satisfying the pharmacophore features subsequently underwent molecular docking to generate preliminary predictions of binding poses and affinities. These filtering steps significantly reduce the chemical space, which is convenient for subsequent computationally intensive steps. MD simulations and supplementary binding free energy calculations were then conducted on the selected complex systems to refine binding mode predictions and quantify ligand-binding energetics with enhanced accuracy. Finally, a subset of candidate molecules exhibiting favorable binding modes and affinities was prioritized for further investigation.

#### 4.1.1. Library Preparation

A Diversity Core Library comprising 75,920 compounds from ChemDiv Inc. (San Diego, CA, USA) was employed for virtual screening. Conformational sampling was performed to constitute the initial dataset (Dataset 1). A maximum of 250 conformations per molecule were generated using the “Conformation Import” protocol implemented in Molecular Operating Environment (MOE, version 2015).

#### 4.1.2. Pharmacophore Modeling and Screening

Binding poses of inhibitors from available PDE1 crystal structures in the Protein Data Bank were used to construct a 3D pharmacophore model. Spatial distribution of substituents and interaction patterns between the ligands and PDE1 were systematically analyzed, identifying key pharmacophore features, including but not limited to aromatic rings, hydrophobic centers, and hydrogen bond donors/acceptors. Molecules in Dataset 1 were subsequently screened against this pharmacophore model, resulting in the reduced Dataset 2.

#### 4.1.3. Molecular Docking and Screening

The Surflex-dock protocol [39] implemented in Tripos Sybyl (version X1.2) was employed for molecular docking of selected molecules in Dataset 2, following the method described in our previous research (see also the Supporting Information) [32,40]. Molecules with favorable docking scores and rational binding modes were retained to constitute Dataset 3.

#### 4.1.4. Molecular Dynamics Simulations

PAINS [35] were excluded from Dataset 3 to minimize false positive compounds arising from nonspecific interactions in enzymatic assays. Then, MD simulations were performed for each ligand-PDE1 complex to equilibrate the binding modes and refine the binding energies. Partial atomic charges of each ligand pose were calculated using the Hartree-Fock method at the 6-31G* level with Gaussian (version 03) [41]. The Antechamber program was then employed to fit the restricted electrostatic potential (RESP) charges and assign atom types and parameters from the general AMBER force field (GAFF). The relevant parameters for the protein were described using the Amber03 force field. Each system was solvated in an 8 Å truncated octahedron TIP3P water box and was neutralized by Na^+^ counterions.

MD simulations were performed using the pmemd.cuda module in the software Amber (version 16.0). Each system was equilibrated and subjected to a 100 ns production run in the *NPT* ensemble with a constant temperature of 300 K and a pressure of 1 atm. Periodic boundary conditions were applied, and long-range electrostatic interactions were processed with the particle mesh Ewald (PME) method [42] using an 8 Å cutoff. All bonds involving hydrogen atoms were constrained using the SHAKE algorithm [43], enabling a time step of 2 fs. Simulations were accelerated using GPU resources (NVIDIA Tesla V100 GPUs, NVIDIA Corporation, Santa Clara, CA, USA). Binding free energies were calculated using the MM-PBSA and MM-GBSA methods based on 100 snapshots extracted from the last 2.5 ns of each trajectory, with default parameter settings (see Supporting Information). Conformational entropy contributions were neglected to reduce the computational cost.

### 4.2. In Vitro Assay for Potential PDE1 Inhibitors

Compounds were purchased from TargetMol Chemicals Inc. (Boston, MA, USA). PDE1 and PDE9 enzymatic activity was assayed as previously reported [24,25]. Briefly, PDE1C (147–531) was incubated with ^3^H-cGMP, as well as tested compounds with concentrations of 10 and 1 μM at room temperature (25 ± 1 °C) for 15 min. The hydrolysis reaction was terminated by sequential addition of 0.2 M zinc sulfate and 0.2 M barium hydroxide. Following high-speed centrifugation at room temperature for 15 min, the supernatant was mixed with Ultima Gold scintillation cocktail, and residual radioactivity was measured using a PerkinElmer 2910 liquid scintillation counter (PerkinElmer, Inc., Waltham, MA, USA). Selectivity was assessed by evaluating the inhibitory activities against PDE9A (181–506) with concentrations of 50 and 10 μM under identical conditions.

## 5. Conclusions

In summary, an integrated workflow combining pharmacophore modeling, molecular docking, and dynamics simulations was developed for the virtual screening of novel PDE1 inhibitors. Twenty-six compounds were subjected to enzymatic assays, resulting in eleven active hits with moderate PDE1 inhibition, with a hit rate of 42%. Notably, nine structurally distinct scaffolds were identified, substantially expanding the chemical diversity of current PDE1 inhibitors. Binding mode and affinity analysis revealed that these inhibitors participated in interactions with both conserved residues (Gln421, Phe424, and Phe392) and unique ones (His373 and Thr271) within the active site. Given that no comprehensive SAR has been established for PDE1 inhibitors, the interaction patterns revealed by our docking and MD analysis—particularly the role of the flexible H-loop region—provide testable hypotheses for future rational design. We believe this integrated strategy will facilitate the discovery of novel PDE1 inhibitors for the treatment of IPF and can be readily adapted to other therapeutic targets. Determination of IC_50_ values across different PDEs and full SAR profiling for the most promising scaffolds will be pursued in our ongoing work.

## Figures and Tables

**Table 1:** Performance metrics of pharmacophore-based virtual screening on the test set.

Parameter	Value
Total number of compounds in the test set (D)	2020
Total number of active compounds in the test set (A)	20
Total number of hit compounds (H_t_)	42
Known active compounds in the hit list (H_a_)	17
Enrichment factor (EF)	40.88 ^1^
GH score	0.51 ^2^

^1^ Calculated according to the equation: EF = (H_a_/H_t_) × (D/A). ^2^ Calculated according to the equation: GH score = (H_a_/A) × [(3A + H_t_)/4H_t_] × [1 − (H_t_ − H_a_)/(D − A)].

**Table 2:** Binding free energies and inhibitory activities against PDE1 of compounds obtained from virtual screening.

No.	Compound ID	Chemical Structure	ΔG_pred_/(kcal·mol^−1^) ^1^	PDE1 Inhibition Rate (%) ^2^	
				at 10 μM	at 1 μM
1	8012-2471	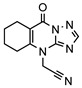	−25.87 ± 1.74	100	18.2
2	6484-0008	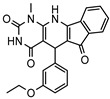	−30.16 ± 2.38	97.3	50.7
3	C200-4020	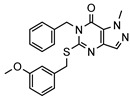	−37.36 ± 1.79	87.9	44.9
4	5223-1356	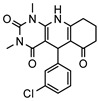	−31.71 ± 2.55	84.5	34.9
5	8020-2441	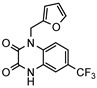	−25.55 ± 2.61	80.9	32.2
6	D245-0568	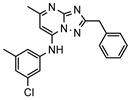	−36.67 ± 2.18	72.4	19.3
7	6484-0032	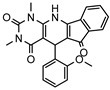	−30.64 ± 2.40	70.2	54.0
8	3389-0956	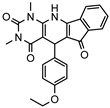	−20.12 ± 2.67	59.4	22.9
9	F279-0419	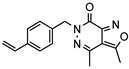	−33.51 ± 1.98	57.5	13.3
10	L397-0566	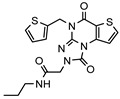	−38.55 ± 2.67	55.5	30.4
11	P300-1479	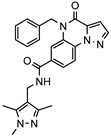	−37.54 ± 2.86	54.0	2.9
12	G652-4613	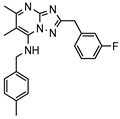	−30.05 ± 2.79	48.5	40.9
13	D116-0357	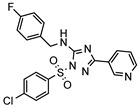	−41.28 ± 2.74	44.8	22.0
14	8020-7630	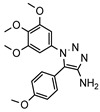	−23.68 ± 2.56	38.3	4.3
15	M652-0610	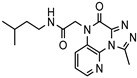	−28.56 ± 2.46	35.7	16.9
16	C200-7927	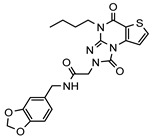	−43.20 ± 2.64	35.5	21.0
17	J108-0587	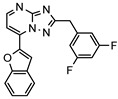	−31.52 ± 2.45	33.1	18.2
18	D245-0478	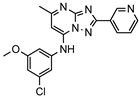	−35.58 ± 3.35	22.6	n.d. ^2^
19	C276-1683	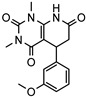	−13.80 ± 1.68	22.1	n.d. ^3^
20	G761-2889	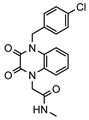	−36.15 ± 2.53	18.4	0
21	8018-0728	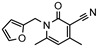	−29.58 ± 2.17	12.8	0
22	G373-3180	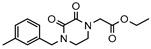	−21.59 ± 2.19	11.1	n.d. ^3^
23	M652-0525	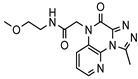	−30.51 ± 2.48	10.5	9.8
24	J006-1371	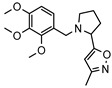	−36.31 ± 2.68	3.2	0
25	8020-0186	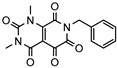	−28.15 ± 2.03	2.6	2.2
26	D679-0170	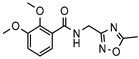	−32.93 ± 2.46	0.4	0

^1^ Predicted data by the MM-GBSA method. ^2^ The previously reported compound (*R*)-1-(*tert*-butyl)-6-((1-(4-chlorophenyl)ethyl)amino)-5-(4-fluorobenzyl)-1,5-dihydro-4H-pyrazolo [3,4-*d*]pyrimidin-4-one was used as a reference inhibitor with an inhibition rate of 50.6% at a concentration of 2.5 nM [15]. ^3^ Not determined.

## Data Availability

The data presented in this study are available within the article and Supplementary Materials.

## Supplementary Materials

The following supporting information can be downloaded at: https://www.mdpi.com/article/10.3390/molecules31101731/s1, Figure S1: The binding modes of representative molecules with PDE1 based on molecular docking; Figure S2. RMSD of the protein backbone relative to the initial structure over the 100 ns trajectory; Table S1: The predicted binding free energies of the 26 purchased compounds and their inhibition ratios against PDE9; Table S2: Hydrogen bond analysis of the inhibitors with PDE1; Table S3: Comparison of chemotypes, potency, and selectivity of the identified and reported hits as PDE1 inhibitors; Table S4: Redocking validation results showing the RMSD values between the crystallographic poses and the docking poses of the five known PDE1 inhibitors.

## Abbreviations

The following abbreviations are used in this manuscript:

CaM	calcium-calmodulin
cAMP	Adenosine 3′,5′-cyclic phosphate
cGMP	Guanosine 3′,5′-cyclic phosphate
GAFF	General AMBER force field
GH	Goodness of Hit
IPF	Idiopathic pulmonary fibrosis
LTP	Long-term potentiation
MAPK	Mitogen-activated protein kinase
MD	Molecular dynamics
MM-PBSA	Molecular Mechanics-Poisson–Boltzmann Surface Area
MM-GBSA	Molecular Mechanics-Generalized Born Surface Area
PDE	Phosphodiesterase
PME	Particle mesh Ewald
RMSD	Root-mean-square deviation
SAR	Structure-activity relationship
TGF-β	Transforming growth factor-β
VSMC	Vascular smooth muscle cell
