# Supplementary material for: Integrated Pharmacophore Modeling, Molecular Docking, and Molecular Dynamics Simulations Accelerate the Discovery of Novel PDE1 Inhibitors with Potential for the Treatment of Idiopathic Pulmonary Fibrosis
Source: Molecules. 2026 May 19;31(10):1731. doi: 10.3390/molecules31101731 (PMC13209513; doi:10.3390/molecules31101731)
Supplement: Supplementary file 1 [file molecules-31-01731-s001.zip › molecules-4291731-supplementary.pdf]

Supporting Information

Integrated Pharmacophore Modeling, Molecular Docking, and Molecular Dynamics Simulations Accelerate the Discovery of Novel PDE1 Inhibitors with Potential for the Treatment of Idiopathic Pulmonary Fibrosis

Xin-Lin Cai [1,#], Zhao-Hang Xue [1,#], Shu-Jin He [1], Wei-Hao Luo [1], Run-Duo Liu [2], Qian Zhou [3,*] and Chen Zhang [1,*]

[1] School of Chemistry and Chemical Engineering, Guangdong Pharmaceutical University, Zhongshan 528458, China; 2112440011@stu.gdpu.edu.cn (X.-L.C.); 2112340005@stu.gdpu.edu.cn (Z.-H.X.); 2112542076@stu.gdpu.edu.cn (S.-J.H.); 2112373045@stu.gdpu.edu.cn (W.-H.L.)

[2] School of Pharmaceutical Sciences, Sun Yat-sen University, Guangzhou 510006, China; liurd3@mail2.sysu.edu.cn (R.-D.L.)

[3] Key Laboratory of Tropical Biological Resources of Ministry of Education and Hainan Engineering Research Center for Drug Screening and Evaluation, School of Pharmaceutical Sciences, Hainan University, Haikou 570228, China

[*] Correspondence: zhouqian@hainanu.edu.cn (Q.Z.); zhangch12020@gdpu.edu.cn (C.Z.)

[#] These authors contributed equally to this work.

Table of Contents:

S1. Figure S1. The binding modes of representative molecules with PDE1 based on molecular docking……………………………………………………………………………………………S3

S2. Figure S2. RMSD of the protein backbone relative to the initial structure over the 100 ns trajectory…………………………………………………………………………………………S10

S3. Table S1. The predicted binding free energies of the 26 purchased compounds and their inhibition ratios against PDE9………………………………………………………………………………S11

S4. Table S2. Hydrogen bond analysis of the inhibitors with PDE1…………………………….….S13
S5. Table S3. Comparison of chemotypes, potency, and selectivity of the identified and reported hits as PDE1 inhibitors……………………………………………………………………………..S14
S6. Table S4. Redocking validation results showing the RMSD values between the crystallographic poses and the docking poses of the five known PDE1 inhibitors……………………………………S15
S7. Details on molecular docking………………………………………………………………….S16
S8. Details on binding free energy calculations…………………………………………………...S17

S1. Figure S1. The binding modes of representative molecules with PDE1 based on molecular docking

(A)

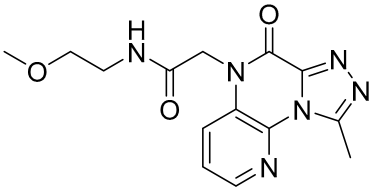

M652-0525

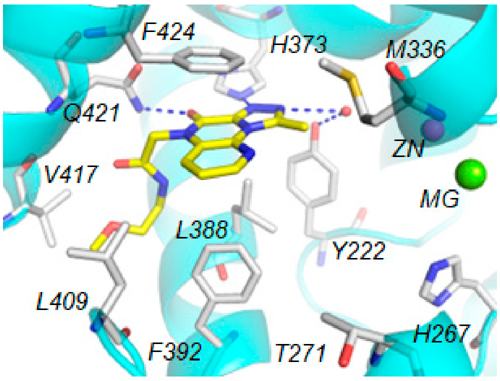

(B)

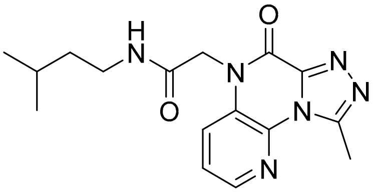

M652-0610

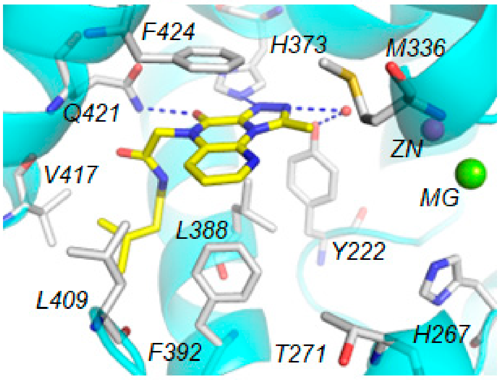

(C)

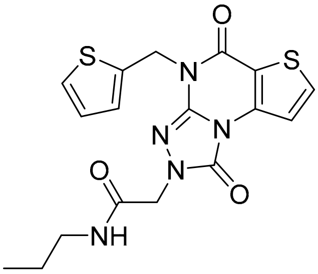

L397-0566

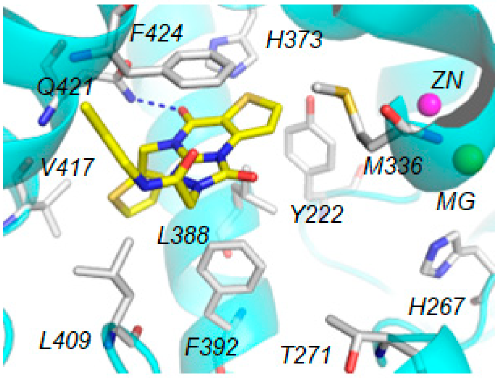

(D)

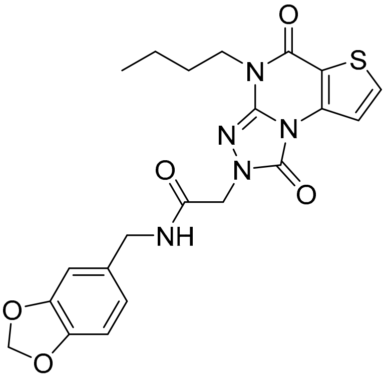

C200-7927

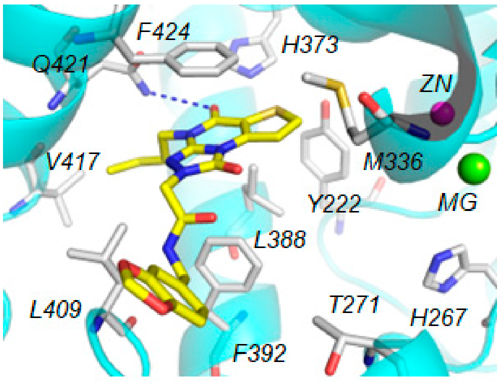

(E)

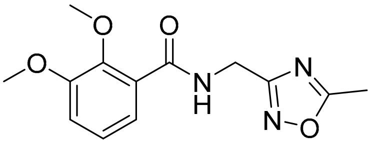

D679-0170

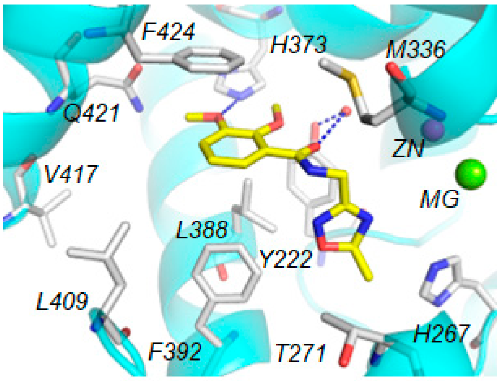

(F)

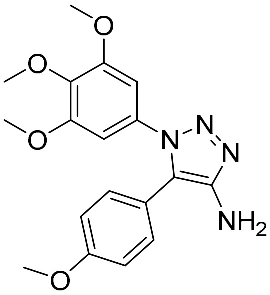

8020-7630

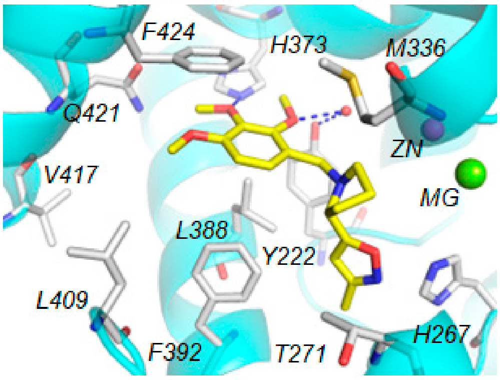

(G)

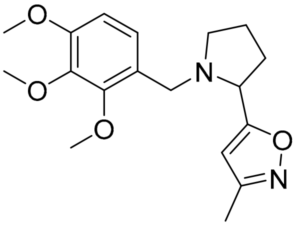

J006-1371

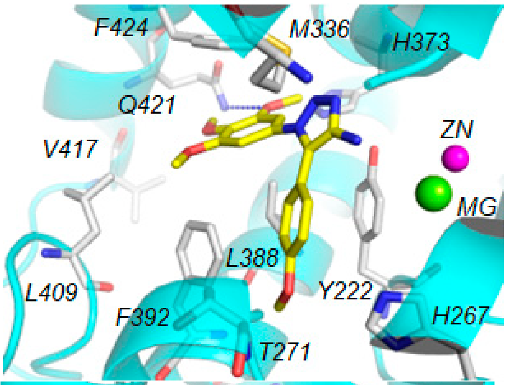

(H)

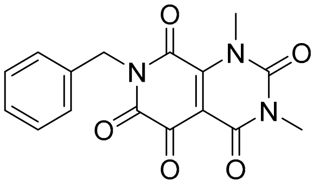

8020-0186

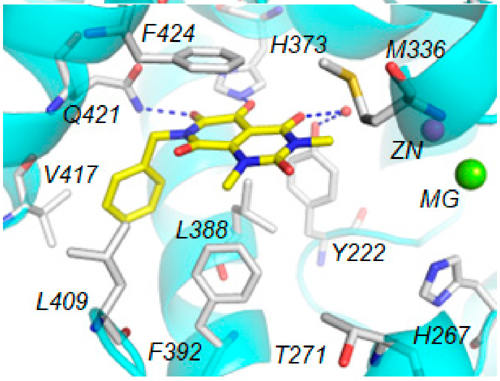

(I)

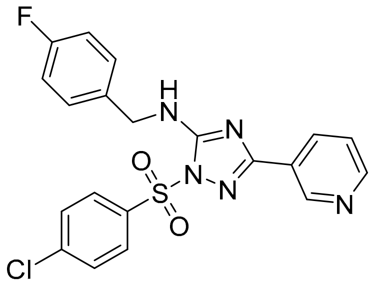

D116-0357

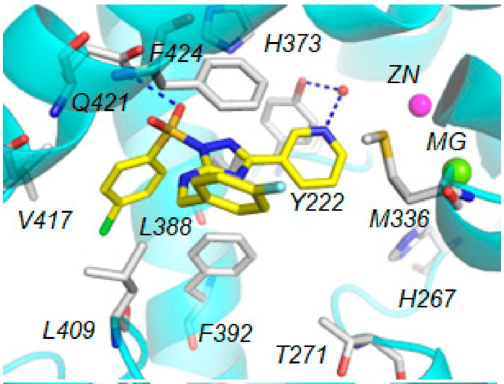

(J)

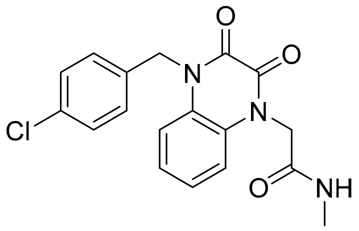

G761-2889

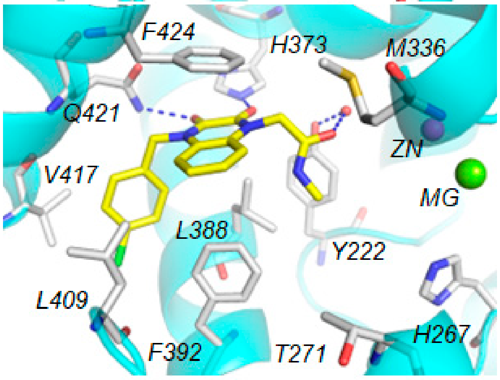

(K)

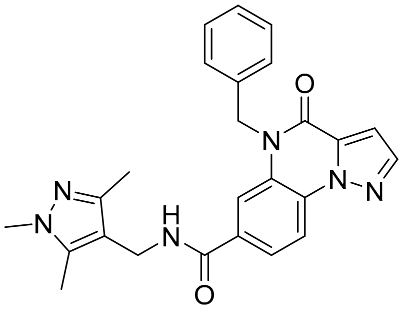

P300-1479

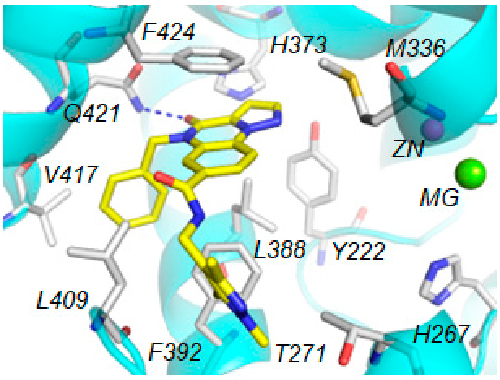

(L)

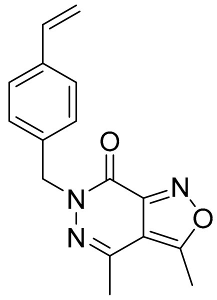

F279-0419

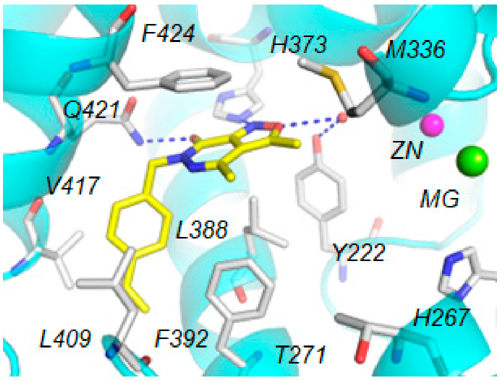

(M)

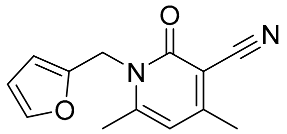

8018-0728

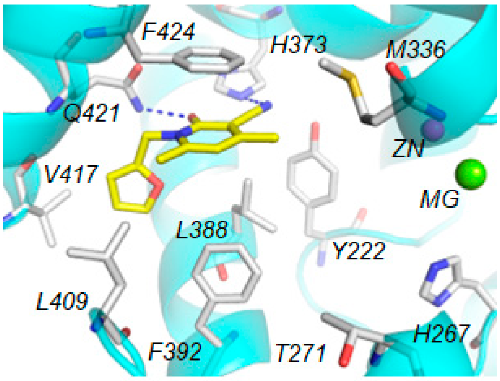

(N)

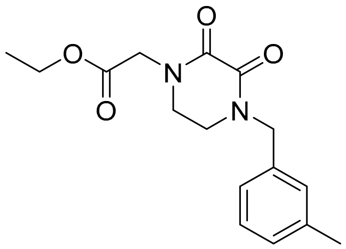

G373-3180

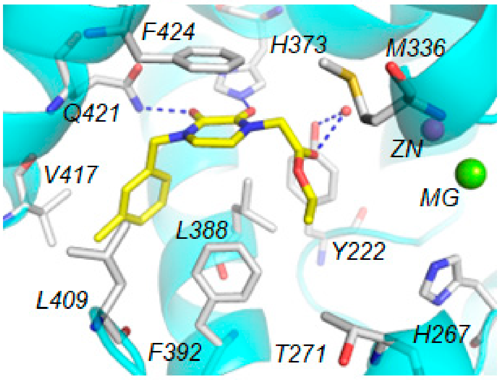

(O)

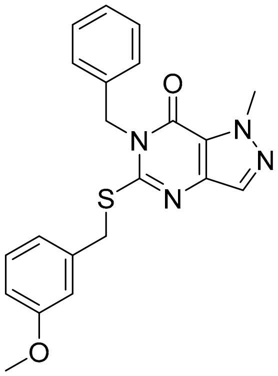

C200-4020

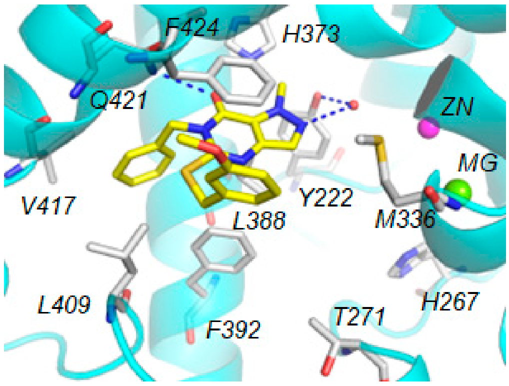

(P)

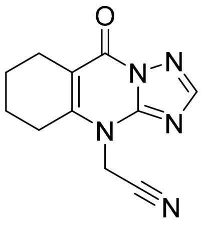

8012-2471

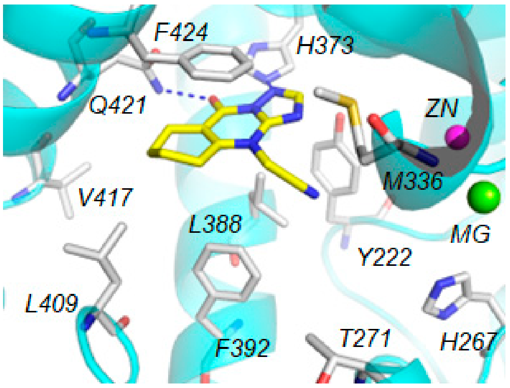

(Q)

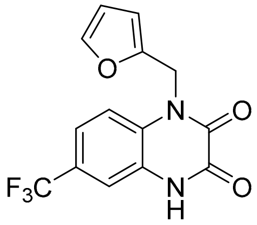

8020-2441

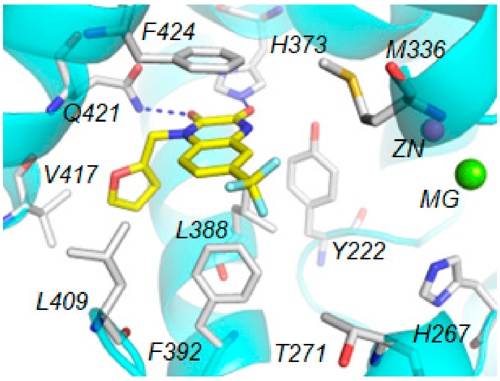

(R)

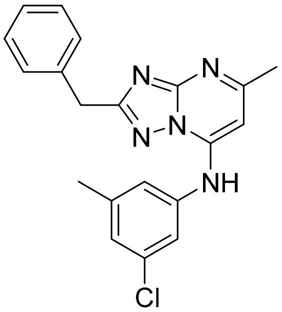

D245-0568

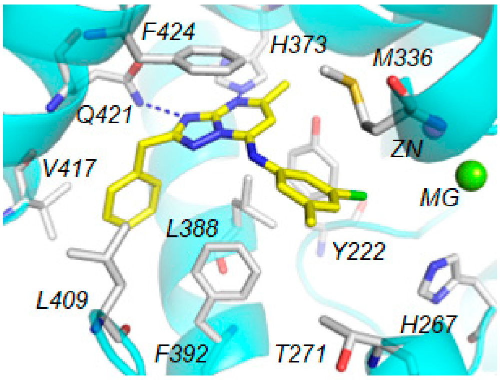

(S)

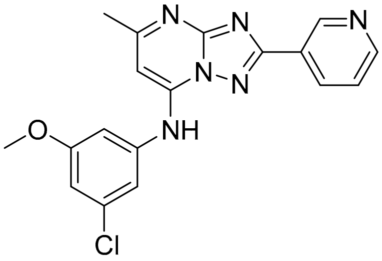

D245-0478

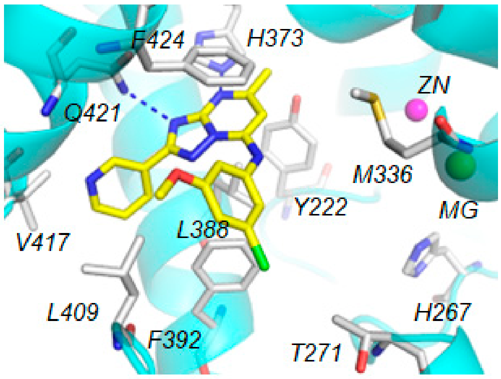

(T)

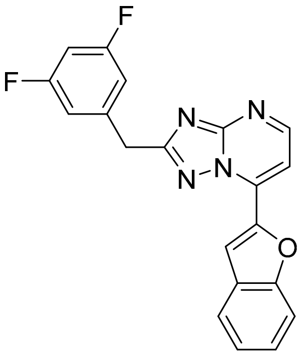

J108-0587

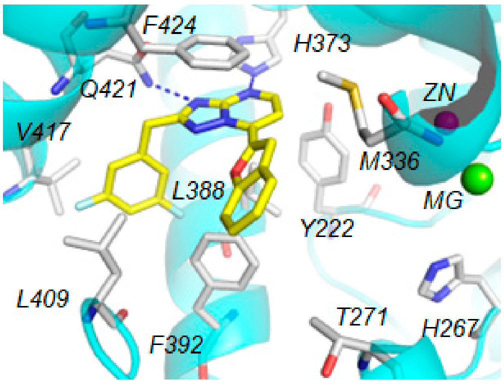

(U)

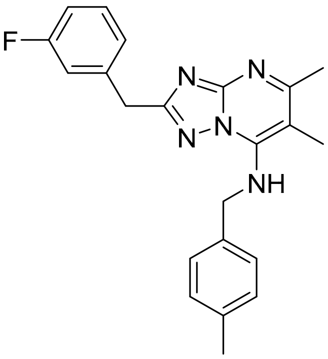

G652-4613

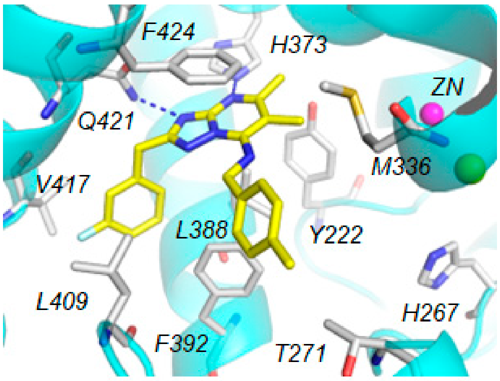

(V)

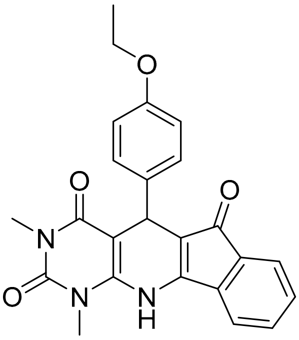

3389-0956

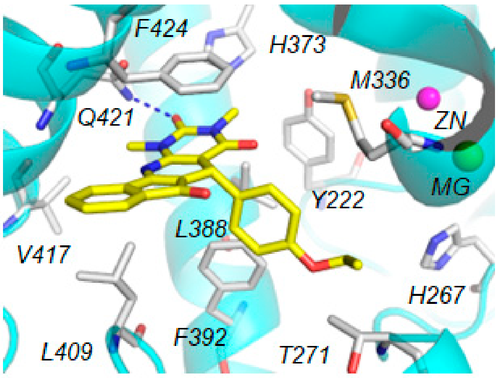

(W)

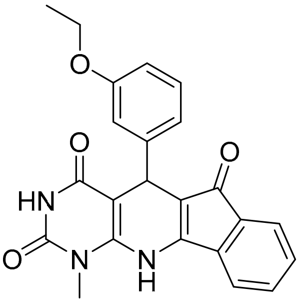

6484-0008

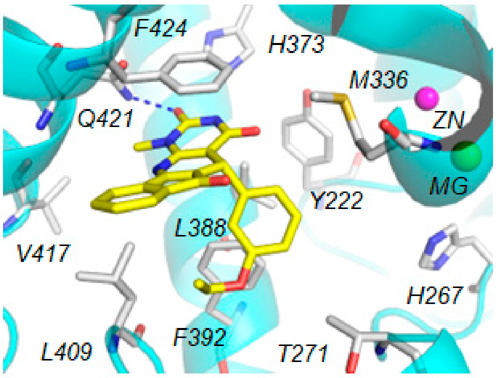

(X)

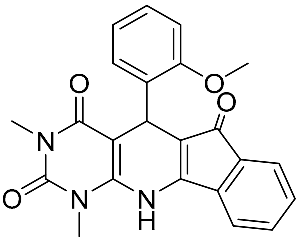

6484-0032

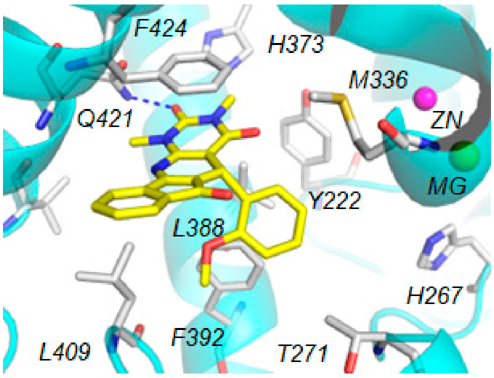

(Y)

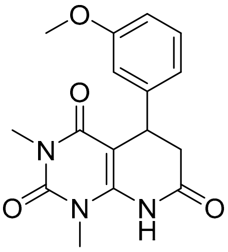

C276-1683

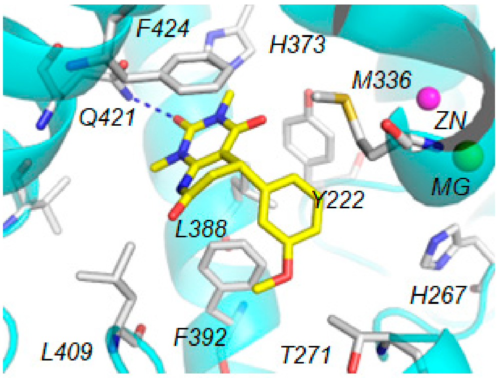

(Z)

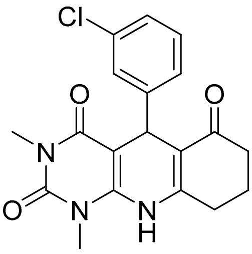

5223-1356

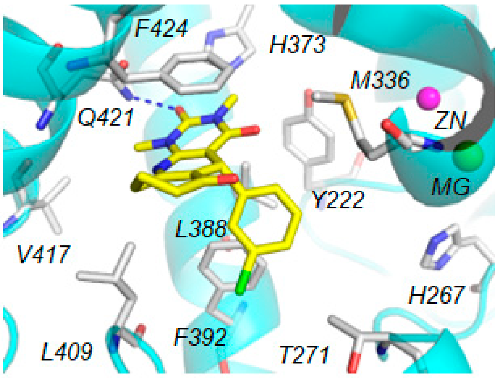

Figure S1. The binding modes of representative molecules with PDE1 based on molecular docking.

S2. Figure S2. RMSD of the protein backbone relative to the initial structure over the 100 ns trajectory.

(A)

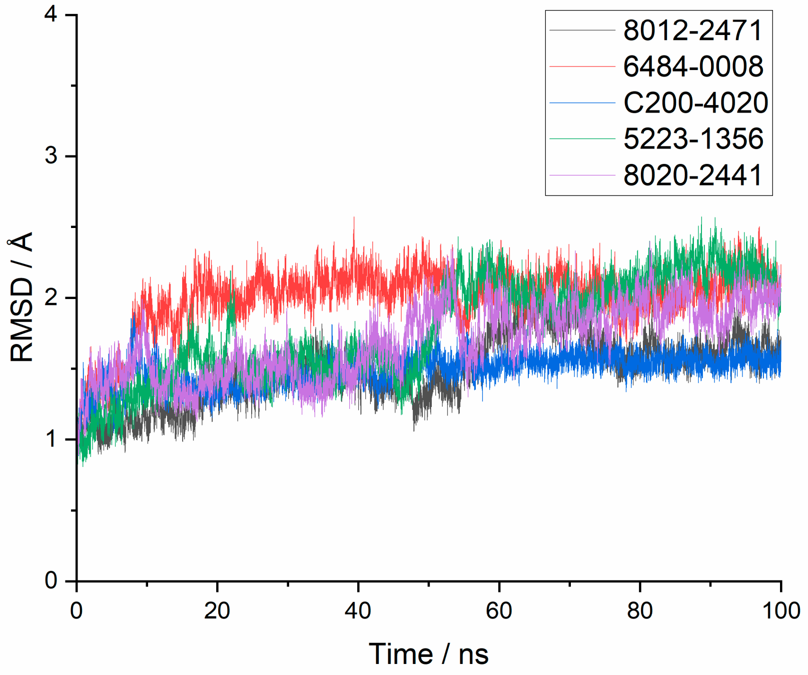

(B)

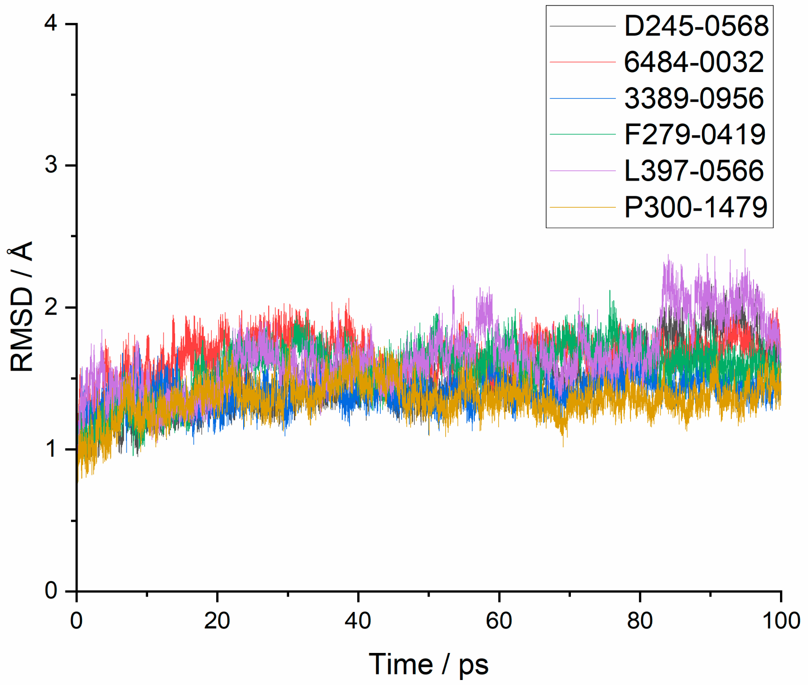

S3. Table S1. The predicted binding free energies of the 26 purchased compounds with PDE1 and their inhibition ratios against PDE9.

Compound ID	ΔG_{pred} [a] (kcal/mol)	ΔG_{pred} [b] (kcal/mol)	PDE9 Inhibition Rate (%) [c]	
			at 50 μM	at 10 μM
8012-2471	−27.42 ± 2.08	−25.87 ± 1.74	11.4	0
6484-0008	−6.46 ± 11.83	−30.16 ± 2.38	40.4	16.6
C200-4020	−40.03 ± 2.26	−37.36 ± 1.79	8.1	2.2
5223-1356	−26.89 ± 4.59	−31.71 ± 2.55	4.9	0
8020-2441	−28.90 ± 2.88	−25.55 ± 2.61	60.1	33.2
D245-0568	−37.35 ± 3.12	−36.67 ± 2.18	13.8	0
6484-0032	−31.85 ± 2.94	−30.64 ± 2.40	22.8	14.8
3389-0956	−24.06 ± 2.55	−20.12 ± 2.67	29.0	11.7
F279-0419	−32.81 ± 3.25	−33.51 ± 1.98	28.4	25.4
L397-0566	−38.35 ± 2.92	−38.55 ± 2.67	30.1	18.6
P300-1479	−39.83 ± 3.36	−37.54 ± 2.86	17.9	15.4
G652-4613	−27.12 ± 3.92	−30.05 ± 2.79	23.1	11.6
D116-0357	−40.46 ± 3.46	−41.28 ± 2.74	n.d. [d]	n.d.
8020-7630	−24.85 ± 2.97	−23.68 ± 2.56	n.d.	n.d.
M652-0610	−27.28 ± 2.90	−28.56 ± 2.46	n.d.	n.d.
C200-7927	−45.48 ± 3.11	−43.20 ± 2.64	n.d.	n.d.
J108-0587	−30.62 ± 3.31	−31.52 ± 2.45	n.d.	n.d.
D245-0478	−35.55 ± 3.27	−35.58 ± 3.35	n.d.	n.d.
C276-1683	−13.55 ± 4.67	−13.80 ± 1.68	n.d.	n.d.
G761-2889	−37.85 ± 3.64	−36.15 ± 2.53	n.d.	n.d.
8018-0728	−30.67 ± 2.66	−29.58 ± 2.17	n.d.	n.d.
G373-3180	−12.69 ± 5.94	−21.59 ± 2.19	n.d.	n.d.
M652-0525	−31.39 ± 2.62	−30.51 ± 2.48	n.d.	n.d.
J006-1371	−4.89 ± 15.06	−36.31 ± 2.68	n.d.	n.d.
8020-0186	−29.85 ± 2.27	−28.15 ± 2.03	n.d.	n.d.
D679-0170	−30.00 ± 3.41	−32.93 ± 2.46	n.d.	n.d.

[a] Binding free energies predicted by MM-PBSA method, based on the last 2.5 ns of the molecular dynamics simulation trajectories.

[b] Binding free energies predicted by MM-GBSA method, based on the last 2.5 ns of the molecular dynamics simulation trajectories.

[c] Inhibition rate against PDE9A (181–506). 1-(5-fluoro-2-(2-morpholinoethoxy)benzylidene)-6-(3-fluorobenzyl)furo[3,4-c]pyridine-3,4(1H,5H)-dione was used as a positive control with 49.6% inhibition ratio at 30 nM concentrations (Li, Z.; Jiang, M.Y.; Liu, R. et al. Discovery of highly potent phosphodiesterase-1 inhibitors by a combined-structure free energy perturbation approach. *Acta Pharm Sin B*. 2024, *14*(12), 5357-5369).

[d] n.d.: not determined.

S4. Table S2. Hydrogen bond analysis of the inhibitors with PDE1.

Compound ID	Fraction forming a hydrogen bond with Gln421 (%) [a]	Bond Parameters [b]		Fraction forming a hydrogen bond with His373 (%) [a]	Bond Parameters [b]	
		Length (Å)	Angle (°)		Length (Å)	Angle (°)
C200-4020	3.1	2.88	161.9	72.8	2.84	158.8
D245-0568	24.4	2.92	149.5	38.0	2.92	158.0
6484-0032	38.7	2.88	162.5	27.7	2.87	156.0
3389-0956	41.8	2.87	156.3	19.6	2.88	160.5
L397-0566	46.8	2.88	159.0	24.7	2.87	149.5
5223-1356	54.4	2.86	162.1	14.6	2.88	154.6
P300-1479	57.1	2.87	163.1	18.4	2.85	151.5
8020-2441	60.8	2.85	158.2	35.7	2.88	151.8
6484-0008	66.9	2.86	163.5	5.0	2.85	148.0
8012-2471	71.0	2.86	159.3	30.7	2.91	149.7
F279-0419	79.8	2.85	159.7	21.3	2.90	148.8

[a] Statistical data from 20 ns MD simulations (4000 frames).

[b] Average values from frames forming a hydrogen bond.

S5. Table S3. Comparison of chemotypes, potency, and selectivity of the identified and reported hits as PDE1 inhibitors.

Method of screening	Number of hits	Chemotypes	PDE1 IC_{50} (µM)	Selectivity	Reference
HTS [a] of internal compound library	1	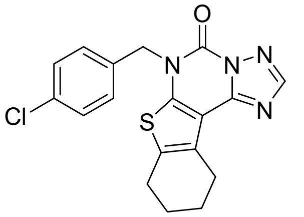	0.460	No selectivity against PDE5/6	[27]
HTS of internal compound library	2	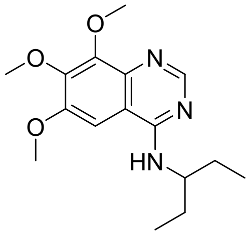	0.219	> 4-fold against PDE10	[33]
		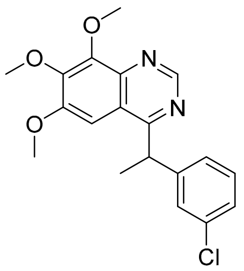	0.130	No selectivity against PDE10	
Virtual Screening	1	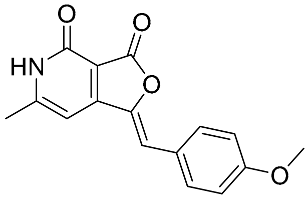	16.8	Not tested	[38]
Virtual Screening	11	10 distinct scaffolds	< 10	> 5-fold against PDE9	This research

[a] High throughput screening.

S6. Table S4. Redocking validation results showing the RMSD values between the crystallographic poses and the docking poses of the five known PDE1 inhibitors.

**PDB ID**	4NPV	4NPW	5B25	5UOY	5UP0
**RMSD (Å)**	1.44	1.23	0.70	1.15	0.45

S7. Details on Molecular Docking

The crystal structure of PDE1 complexed with an inhibitor (PDB ID: 5B25) was used in this study. Molecular docking was conducted using Surflex-dock, a component of Tripos Sybyl (version X2.0, Tripos Software, Inc., El Cerrito, CA). Hydrogen atoms were added, and the ionizable residues were protonated to reflect neutral pH conditions. The protomol represents a group of molecular fragments selected from CH_4, C=O, and N−H, and was placed in PDE1's binding pocket by identifying empty 1 Å voxels between marked residues using the bound ligand as the reference. It was then automatically optimized locally by 3D Gaussian smoothing for placement, with high-scoring fragments retained, constituting the docking site. The parameters proto_thresh and proto_bloat indicate the extent to which the protomol can be buried within the protein and the permissible outward extension beyond the cavity, respectively. The proto_thresh was set to 0.5, while the proto_bloat was designated as 0. After preparing the protomol, molecular docking was conducted. The parameter of "Maximum Number of Poses per Ligand" was set to 10, resulting in at most 10 top-ranked docked conformations retained. CScore calculations were performed to obtain docking scores between each conformation and PDE1. All designed molecules were docked to the prepared PDE1 protein, and the top-ranked molecules possessing both higher docking scores and suitable binding patterns were selected for subsequent simulations, followed by binding free energy calculations.

S8. Details on binding free energy calculations

In light of the MM-PBSA and MM-GBSA method, the binding free energy (ΔG_{bind}) can be calculated by the following Eq. S1, where the free energies of complex, receptor and ligand are represented by G_{comp}, G_{rec} and G_{lig}, respectively.

$$\Delta G_{bind} = G_{comp} - G_{rec} - G_{lig} \quad (S1)$$

The binding free energy of each system was evaluated as the sum of the MM energy (E_{MM}), the solvation free energy (G_{solv}), and the entropy contribution (S), respectively, as described in Eq. S2.

$$\Delta G_{bind} = \Delta E_{MM} + \Delta G_{solv} - T\Delta S \quad (S2)$$

ΔE_{MM} is the gas phase interaction energy, which can be decomposed into $E_{MM,comp}$, $E_{MM,rec}$ and $E_{MM,lig}$. Solvation free energy is evaluated by the sum of the electrostatic solvation free energy (ΔG_{PB}) and nonpolar solvation free energy (ΔG_{np}), resulting in Eq. S3.

$$\Delta G_{solv} = \Delta G_{PB} + \Delta G_{np} \quad (S3)$$

ΔG_{PB} was calculated by the Poisson Boltzmann (PB) Eq, whereas ΔG_{np} was calculated according to Eq. S4. The default parameters were adopted, with $\gamma = 0.0072$ kcal/(Å^2) and $b = 0$ kcal/mol.

$$\Delta G_{np} = \gamma \, SASA + b \quad (S4)$$

For a compromise between efficiency and accuracy, the entropy contribution term ($-T\Delta S$) was omitted for ΔG_{bind} in Eq. S2, since the calculations of the entropy contribution are extremely time-consuming for large protein-ligand systems.

The MM-PBSA and MM-GBSA calculations were performed using the MMPBSA.py module implemented in Amber 16. The protein was described using the ff14SB force field, and ligand parameters were generated using the general AMBER force field (GAFF) with AM1-BCC charges assigned by the antechamber module. 100 snapshots were extracted evenly from the equilibrated production trajectory (last 2.5 ns) for each ligand–protein complex. For MM-PBSA, the Poisson–Boltzmann (PB) method was employed with the igb = 2 radii set; for MM-GBSA, the Generalized Born (GB) model (igb = 2) was used. The interior and exterior dielectric constants were set to 1 and

80, respectively; the ionic strength was set to 0.150 M to approximate physiological conditions; and the surface tension was set to 0.0072 kcal/(mol·$Å^2$) for the nonpolar solvation term.
